# Antioxidant and Anti-Inflammatory Effects of Rhei Rhizoma and Coptidis Rhizoma Mixture on Reflux Esophagitis in Rats

**DOI:** 10.1155/2016/2052180

**Published:** 2016-04-27

**Authors:** O Jun Kwon, Min Yeong Kim, Sung Ho Shin, Ah Reum Lee, Joo Young Lee, Bu-il Seo, Mi-Rae Shin, Hyun Gyu Choi, Jeong Ah Kim, Byung Sun Min, Gyo-Nam Kim, Jeong Sook Noh, Man Hee Rhee, Seong-Soo Roh

**Affiliations:** ^1^Kyeoungbuk Institute for Regional Program Evaluation, Gyeongbuk TP, 27 Sampoong-ro, Gyeongsan, Gyeongsangbuk-do 38542, Republic of Korea; ^2^College of Korean Medicine, Daegu Haany University, Gyeongsan 38610, Republic of Korea; ^3^College of Pharmacy, Research Institute of Pharmaceutical Sciences, Kyungpook National University, Daegu 41566, Republic of Korea; ^4^College of Pharmacy, Catholic University of Daegu, Gyeongsangbuk-do 38430, Republic of Korea; ^5^Department of Food Science and Biotechnology, Kyungnam University, Gyeongsangnam-do 51767, Republic of Korea; ^6^Department of Food Science & Nutrition, Tongmyong University, Busan 48520, Republic of Korea; ^7^College of Veterinary Medicine, Kyungpook National University, Daegu 41566, Republic of Korea

## Abstract

The purpose of this study was to investigate the antioxidant and anti-inflammatory effects of the combined extract of Rhei rhizoma and Coptidis rhizoma (RC-mix) in experimental model of acute reflux esophagitis. The antioxidant activity was assessed by* in vitro* 2,2-diphenyl-1-picrylhydrazyl (DPPH) and 2,2′-azino-bis(3-ethylbenzothiazoline-6-sulfonic acid) (ABTS) assays. RC-mix was given at 100, 200, and 400 mg/kg body weight 2 h prior to induction of reflux esophagitis (RE). After 5 h, the effects of RC-mix treated rats were compared with those of normal and control rats. The representative flavonoid contents of RC-mix, such as sennoside A, epiberberine, coptisine, palmatine, and berberine, were detected using HPLC. The elevated esophageal mucosa damage was markedly ameliorated by RC-mix treatment in a dose-dependent manner. Furthermore, the administration of RC-mix reduced the increase of serum reactive oxygen species (ROS) and peroxynitrite (ONOO^−^). The improvement of superoxide dismutase (SOD) and heme oxygenase-1 (HO-1) levels were marked in the group given RC-mix. Moreover, the elevation of inflammatory mediators and cytokines by nuclear factor-kappa B (NF-*κ*B) activation in control rats decreased by RC-mix pretreatment. These results indicate that RC-mix treatment reduces the pathological states of esophagitis* via* regulating NF-*κ*B mediated inflammation related to oxidative stress.

## 1. Introduction

Gastroesophageal reflux disease (GERD) includes a wide range of reflux disease, from intermittent symptoms like acid regurgitation or heartburn to endoscopic reflux esophagitis and Barrett's esophagus [1]. In 2005, the percentage of GERD prevalence in Eastern Asia was 2.5%–4.8%, whereas, in the Western world, it was much higher, about 10%–20% [2]. Thereafter, from 2005 to 2010, it reached the prevalence of 5.2%–8.5% in Eastern Asia and of 6.3%–18.3% in Southeast and Western Asia [3]. The epidemiology of GERD has shown that it is caused by an excessive exposure to gastric contents such as gastric acid, pepsin, trypsin, and bile acids in earlier days [4]. So, a management of GERD requires lifestyle modification, medical therapy like antacids, histamine-receptor antagonists, or proton-pump inhibitors, and surgical therapy [5]. However, despite their well-known healthy effects, these therapies could determine relapse, incomplete mucosal healing, the development of severe complications, and various adverse effects because of the long-term use [6, 7]. Recent studies have reported that oxidative stress has a more important role than acids in the pathogenesis of reflux esophagitis (RE) [8, 9]. The esophageal mucosa is formed by stratified squamous epithelium that consists of 20 to 30 layers of cells. The esophageal mucosa is in a state of continuous exposure to potentially damaging endogenous and exogenous factors. For instance, the gastric acid combined with even small amounts of pepsin causes a potent damage of the mucosal barrier, resulting in increased hydrogen ion permeability, mucosal morphologic changes, and local hemorrhage [10]. The development of reflux esophagitis too, at a cellular level, is due to hydrogen ion diffusion into mucosa, leading to tissue acidification and necrotic damage [11]. All these injuries trigger a series of cellular infiltrations and cytokine release that result in an inflammatory response and damage in the esophageal tissue. Various inflammatory and immune cells including macrophages, neutrophils, dendritic cells, and lymphocytes are activated and generated reactive oxygen species (ROS). Overproduction of ROS can contribute to the immediate development of inflammatory process. Administration of antioxidants acting as free radical scavengers has been found to prevent esophageal mucosal damage by blocking the free radicals [12].

At present, natural products from plant with antioxidant activities have been highlighted as promising sources for treating the inflammation. Rhei rhizoma (RR, Dahuang in Korean medicine) is one of the traditional herbal medicines widely cited in Chinese, Korean, and Japanese pharmacopoeias for its several biological effects, such as purgative, antipyretic, anti-inflammatory, antiangiogenic, and antineoplasmic activities [13]. It has been reported that sennoside A represents one of the more important components of RR and it becomes rheinanthrone, when transformed into its active metabolite. It exerts a protective effect against oxidative stress-related endothelial cell injury [14].* Coptis chinensis* too is a widely used herb in traditional Korean medicine that have attracted much attention because of its multiple pharmacological effects, such as antibacterial, antiviral, anticancer, and antioxidative effects [15, 16]. Berberine, a primary component of Coptidis rhizoma, exerts a potent anti-inflammatory action in various disease [17, 18]. According to these reports, RC-mix may regulate effectively the inflammation of RE against oxidative stress. However, virtually no studies have investigated its chemical profiling and pharmacological activity in reflux-induced esophagitis. Therefore, we investigated the effects of RC-mix on rats with reflux esophagitis to examine its preventive effect against oxidative stress-related inflammation.

## 2. Materials and Methods

### 2.1. Materials. 

The protease inhibitor mixture solution and ethylenediaminetetraacetic acid (EDTA) were purchased from Wako Pure Chemical Industries, Ltd. (Osaka, Japan). Phenylmethylsulfonyl fluoride (PMSF) was purchased from Sigma Chemical Co. (St. Louis, MO, USA). 2′,7′-Dichlorofluorescein diacetate (DCF-DA) was obtained from Molecular Probes (Eugene, OR, USA). The pierce bicinchoninic acid (BCA) protein assay kit was obtained from Thermo Scientific (Rockford, IL, USA). ECL Western Blotting Detection Reagents and pure nitrocellulose membranes were supplied by GE Healthcare (Piscataway, NJ, USA). Rabbit polyclonal antibodies against nuclear factor-kappa B p65 (NF-*κ*Bp65; 1 : 1,000, SC-372), nuclear factor-erythroid 2-related factor 2 (Nrf-2; 1 : 1,000, SC-7228), heme oxygenase-1 (HO-1; 1 : 1,000, SC-10789), superoxide dismutase (SOD; 1 : 1,000, SC-11407), and catalase (1 : 1,000, SC-50508); goat polyclonal antibodies against tumor necrosis factor-*α* (TNF-*α*; 1 : 1,000, SC-1351) and interleukin-6 (IL-6; 1 : 1,000, SC-1266); mouse monoclonal antibodies against cyclooxygenase-2 (COX-2; 1 : 1,000, SC-19999), inducible nitric oxide synthase (iNOS, 1 : 1,000, SC-7271), phosphor-inhibitory kappa B alpha (p-I*κ*B*α*; 1 : 1,000, SC-8404) histone (1 : 1,000, SC-8030), and *β*-actin (1 : 1,000, SC-4778) were purchased from Santa Cruz Biotechnology, Inc. (Santa Cruz, CA, USA). Rabbit anti-goat (1 : 3,000, SC-2774), goat anti-rabbit (1 : 5,000, SC-2004), and goat anti-mouse (1 : 5,000, SC-2005) immunoglobulin G (IgG) horseradish peroxidase- (HRP-) conjugated secondary antibodies were acquired from Santa Cruz Biotechnology, Inc. (Santa Cruz, CA, USA). All other chemicals and reagents used were of the analytical grade commercially available (Sigma Aldrich Co., Ltd., USA).

### 2.2. Plant Materials

Rhei rhizoma (roots of* Rheum tanguticum* Maxim.) and Coptidis rhizoma (roots of* Coptis chinensis* Franch.) were purchased from Ominherb Co. (Yeongcheon, Korea). A voucher herbarium specimen has been deposited at the Herbarium of Daegu Haany University and was identified by Professor S. S. Roh, the herbarium leader of Daegu Haany University. Dried slices of Rhei rhizoma (15 g) and Coptidis rhizoma (15 g) mixture (RC-mix) boiled with distilled water (300 mL) at room temperature for 2 h and the solvent was evaporated* in vacuo* to obtain powder with a yield of 14.2%, by weight, of the original RC-mix.

### 2.3. Analysis of Rhei Rhizoma and Coptidis Rhizoma Mixture by HPLC Chromatogram

**Figure 1:**
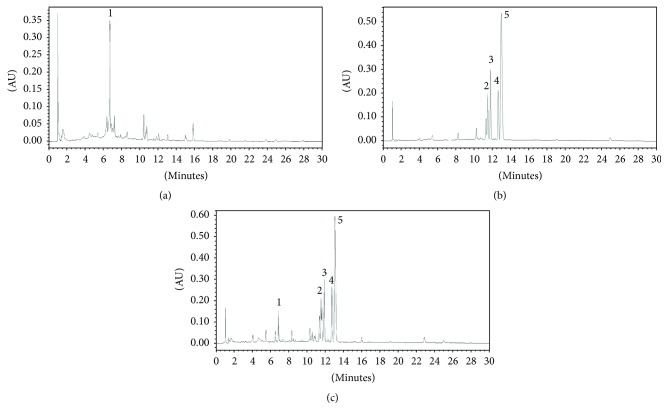
HPLC chromatogram of RC-mix extract (1 mg/mL) detected 254 nm. Signals 1–5 identified to be sennoside A, epiberberine, coptisine, palmatine, and berberine in regular sequence. RC-mix, a water extract of Rhei rhizoma and Coptis rhizoma mixture.

The water extract of Rhei rhizoma and Coptidis rhizoma mixture (1 mg) was dissolved in 1 mL of 50% methanol with multi-vortexing. We injected 50 *μ*L of the sample into a reverse-phase HPLC using a ZORBAX Eclipse XDB-C18, analytical 4.6 × 150 mm, 5 microns, with a column temperature of 25°C. Mobile phase component A is methanol and B is water (10 mM 1-hexanesulfonic acid sodium). The gradient conditions were as follows: 15% A, 0 min, 50% A, 15 min, and 30% A, 30 min. The flow rate was 2.0 mL/min. The UV absorbance from 254 nm was monitored using an Agilent 1200 series with an 2998 Photodiode Array Detector from Waters Co. (Manchester, UK). All peaks were assigned by carrying out coinjection tests with authentic samples and comparing them with the UV spectral data. The major components of Rhei rhizoma and Coptidis rhizoma were sennoside A, epiberberine, coptisine, palmatine, and berberine. Sennoside A was detected from Rhei rhizoma and epiberberine, coptisine, palmatine, and berberine were detected from Coptidis rhizoma. The measurement was repeated three times. Representative HPLC results are illustrated in Figure 1.

### 2.4. Experimental Animals and Treatment

Animal experiments were carried out according to the “Guidelines for Animal Experimentation” approved by the Ethics Committee of the Daegu Haany University (Approval number 2015-055). Six-week-old male Sprague-Dawley rats were purchased from Samtako (Osan, Korea). Rats were maintained under a 12 h light/dark cycle and housed at a controlled temperature (23 ± 1°C) and humidity (about 55%). After adaptation (1 week), a total of 30 SD rats were randomly divided into 5 groups (*n* = 6 per group). The rats were fasted for 18 h prior to surgical procedures and kept in raised mesh-bottom cages to prevent coprophagy but were provided free access to water. The rats were anaesthetized with an injection of Zoletil 0.75 mg/kg (Virbac S. A. France). A midline laparotomy was performed to expose the stomach; both the pylorus and the transitional junction between the forestomach and the corpus were exposed and then ligated with a 2-0 silk thread without a pyloric ring, employing the method originally proposed by Omura et al. [19]. The vagus nerves were left intact. Group one included the normal rats (N). Group two included the RE control rats (Veh). Group three included the RC-mix 100 mg/kg (RC100). Group four included the RC-mix 200 mg/kg (RC200). Group five included the RC-mix 400 mg/kg (RC400). The normal and RE control rat groups were given water, while the other groups were orally given RC-mix at a dose of 100, 200, and 400 mg/kg body weight. Based on our previous experiment, the maximum concentration of RC-mix was determined as 400 mg/kg [20]. The administration of water or RC-mix extract in rats was provided using a stomach tube only one time 2 h before abdominal surgery. The rats in all groups were sacrificed 5 h after the surgery. The entire esophagus was removed immediately and examined for gross mucosal injury. The esophageal tissue was immediately frozen in liquid nitrogen, and blood samples were collected by a vena cava puncture from anesthetized rats. Subsequently, the esophagus and serum were kept at −80°C until analysis.

### 2.5. DPPH Radical Scavenging Activity of RC-Mix Extract

Antioxidant activity determination of RC-mix extract was performed by the DPPH radical scavenging according to the method of Hatano et al. [21]. The reduction of the stable purple free radical DPPH to the yellow hydrazine is achieved by trapping the unpaired electrons, and the degree of discoloration indicates the scavenging activity of samples [22]. 100 *μ*L of an ethanolic solution of RC-mix extract (blank: 100 *μ*L of ethanol) was added to 100 *μ*L of an ethanolic solution of DPPH (60 *μ*M) using 96-well microtitre plate. The ascorbic acid (standard sample) and RC-mix extract were prepared for eight concentrations (1, 2.5, 5, 10, 25, 50, 100, and 200 *μ*g/mL). After mixing gently and leaving to stand for 30 min at room temperature, the optical density was determined using a Microplate Reader, model infinite M200 PRO (Tecan, Austria). The mixture was measured spectrophotometrically at 540 nm. The antioxidant activity of each sample was expressed in terms of the IC_50_ (micromolar concentration required to inhibit DPPH radical formation by 50%, calculated from the log-dose inhibition curve). The radical scavenging activity was calculated as a percentage using the following equation:

(1) DPPH  radical  scavenging  activity%=1−AsampleAblank×100.

### 2.6. ABTS Radical Scavenging Activity of RC-Mix Extract

ABTS radical scavenging activity of the different extracts was measured according to the modified method of Re et al. [23]. ABTS stock solution was dissolved in water to a 7.4 mM concentration. The ABTS radical cation (ABTS) was produced by reacting ABTS stock solution with 2.45 mM potassium persulfate and allowing the mixture to stand for 14 h at room temperature in the dark. The ABTS solution was diluted with ethanol to obtain an absorbance of 0.70 ± 0.02 at 750 nm. After adding 95 *μ*L of diluted ABTS solution (*A*_750 nm_ = 0.70 ± 0.02) to 5 *μ*L of sample, the mixture was left at room temperature for 15 min in the dark. The absorbance at 750 nm was measured using a Microplate Reader, model infinite M200 PRO (Tecan, Austria). The blank was prepared in the same manner, except distilled water was used instead of the sample. The radical scavenging activity was calculated as a percentage using the following equation:

(2) ABTS  radical  scavenging  activity%=1−AsampleAblank×100.

### 2.7. Esophageal Lesion Score

The rat esophagus was cut with scissors in longitudinal direction from the gastroesophageal junction to the pharynx after sacrifice. The inner mucus was washed away with 0.9% NaCl and laid out on paper. Thereafter, the dissected esophagus was photographed with an optical digital camera (Sony, Tokyo, Japan) and analyzed using the i-Solution Lite software program. The gross mucosal damage ratio was calculated as follows:

(3) The  gross  mucosal  damage  ratio%=width  of  area  with  esophageal  mucosal  damagemm2width  of  total  area  of  esophagusmm2×100.

### 2.8. Measurement of ROS Level in the Serum

The ROS levels were measured by employing the method of Ali et al. [24]. 25 mM DCF-DA was added to the serum. After incubation for 30 min, the changes in fluorescence values were determined at an excitation wavelength of 486 nm and emission wavelength of 530 nm.

### 2.9. Measurement of Peroxynitrite Level in the Serum

The peroxynitrite (ONOO^−^) level was assessed by a modified Kooy's method with minor modifications [25], which involves the monitoring of highly fluorescent rhodamine 123, which is rapidly produced from nonfluorescent dihydrorhodamine (DHR) 123 in the presence of ONOO^−^. And its final fluorescent intensities remained unchanged over time. Serum was added to the rhodamine buffer. In brief, the rhodamine buffer (pH 7.4) consisted of 50 mM sodium phosphate dibasic, 50 mM sodium phosphate monobasic, 90 mM sodium chloride, 5 mM potassium chloride, and 5 mM diethylenetriamine penta-acetic acid. The final DHR 123 concentration was 5.0 *μ*M. Five minutes after treating with or without the addition of authentic ONOO^−^, the background and final fluorescent intensities of the samples were measured. The assay buffer was prepared prior to use and placed on ice. The fluorescence intensity of the oxidized DHR 123 was measured with a microplate fluorescence reader, model infinite M200 PRO (Tecan, Austria), at 485 nm excitation and 535 nm emission. The results were expressed as the inhibition level of oxidation of DHR 123 and calculated from the final fluorescence intensity minus background fluorescence intensity.

### 2.10. Preparation of Cytosol and Nuclear Fractions

Protein extraction was performed according to the method of Komatsu with minor modifications [26]. Esophageal tissues for cytosol fraction were homogenized with ice-cold lysis buffer A (250 mL) containing 10 mM HEPES (pH 7.8), 10 mM KCl, 2 mM MgCl_2_, 1 mM DTT, 0.1 mM EDTA, 0.1 mM PMSF, and 1,250 *μ*L protease inhibitor mixture solution. The homogenate incubated at 4°C for 20 min. And then 10% NP-40 was added and mixed well. After centrifugation (13,400 ×g for 2 min at 4°C) using Eppendorf 5415R (Hamburg, Germany), the supernatant liquid (cytosol fraction) was separated by new e-tube. The left pellets were washed twice by buffer A and the supernatant was discarded. Next, the pellets were suspended with lysis buffer C (20 mL) containing 50 mM HEPES (pH 7.8), 50 mM KCl, 300 mM NaCl, 1 mM DTT, 0.1 mM EDTA, 0.1 mM PMSF, 1% (v/v) glycerol, and 100 *μ*L protease inhibitor mixture solution suspended and incubated at 4°C for 30 min. After centrifugation (13,400 ×g for 10 min at 4°C), the nuclear fraction was prepared to collect the supernatant. Both cytosol and nuclear fractions were kept at −80°C before the analysis.

### 2.11. Immunoblotting Analyses

For the estimation of Nrf-2, NF-*κ*Bp65, and histone, 10 *μ*g of protein from each nuclear fraction was electrophoresed through 8–10% sodium dodecyl sulfate polyacrylamide gel (SDS-PAGE). Separated proteins were transferred to a nitrocellulose membrane, blocked with 5% (w/v) skim milk solution for 1 h, and then incubated with primary antibodies (Nrf-2, NF-*κ*Bp65, and histone) and overnight at 4°C. After the blots were washed, they were incubated with anti-rabbit or anti-mouse IgG HRP-conjugated secondary antibody for 1 h at room temperature. In addition, 10–15 *μ*g protein of each postnuclear fraction of SOD, catalase, HO-1, p-I*κ*B*α*, COX-2, iNOS, TNF-*α*, IL-6, and *β*-actin was electrophoresed through 8–15% SDS-PAGE. Each antigen-antibody complex was visualized using ECL Western Blotting Detection Reagents and detected by chemiluminescence with Sensi-Q 2000 Chemidoc (Lugen Sci Co., Ltd., Gyeonggi-do, Korea). Band densities were measured using ATTO Densitograph Software (ATTO Corporation, Tokyo, Japan) and quantified as the ratio to histone or *β*-actin. The protein levels of the groups are expressed relative to those of the normal rat (represented as 1).

### 2.12. Statistical Analysis

The data are expressed as the mean ± SEM. Significance was assessed by one-way analysis of variance (ANOVA) followed by Dunnett's multiple comparison test using SPSS version 22.0 software (SPSS Inc., Chicago, IL, USA). Values of *p* < 0.05 were considered significant. Also, simple regression analysis was performed to investigate the correlation between DPPH radical scavenging and ABTS radical scavenging using the Microsoft Excel 2010 statistical package.

## 3. Results

### 3.1. Compositional Contents Analysis of RC-Mix Extract Using HPLC Chromatogram

Representative HPLC results are illustrated in Figure 1. The ratio of each flavonoid was as follows: Rhei rhizoma and Coptidis rhizoma mixture: 3.14% sennoside A, 8.08% epiberberine, 7.92% coptisine, palmatine 8.89%, and berberine 28.96%. The data showed that the alkaloid present at the highest amount of RC-mix was berberine. The next most abundant ones were palmatine and coptisine.

### 3.2. DPPH Radical Scavenging Activity and ABTS Radical Scavenging Activity

**Figure 2:**
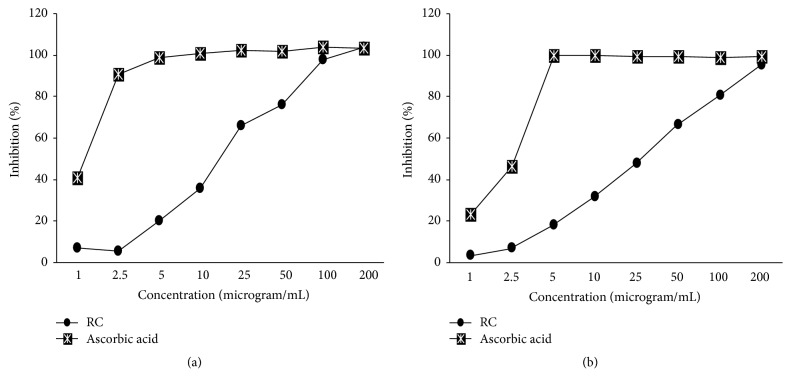
DPPH radical scavenging activity (a) and ABTS radical scavenging activity (b) of RC-mix, water extract of Rhei rhizoma and Coptis rhizoma mixture. Each experiment was run in triplicate.

In this study,* DPPH* and* ABTS radical scavenging activity* were performed to determine and confirm antioxidant activity of RC-mix. IC_50_ (*μ*g/mL) represents half maximal concentration of tested compounds to scavenge DPPH and ABTS radical. As shown in Figure 2(a), the IC_50_ of DPPH radical scavenging activity of RC-mix was found at 17.74 ± 2.14 *μ*g/mL and the IC_50_ value of ascorbic acid (positive control) as a positive control was 1.28 ± 0.04 *μ*g/mL. The calculated IC_50_ value of RC-mix against the ABTS^*+*^ radical was determined to be 27.60 ± 0.94 *μ*g/mL and the IC_50_ value of ascorbic acid (positive control) as a positive control was 2.20 ± 0.02 *μ*g/mL (Figure 2(b)).

### 3.3. Gross Mucosal Damage in the Esophagus 

**Figure 3:**
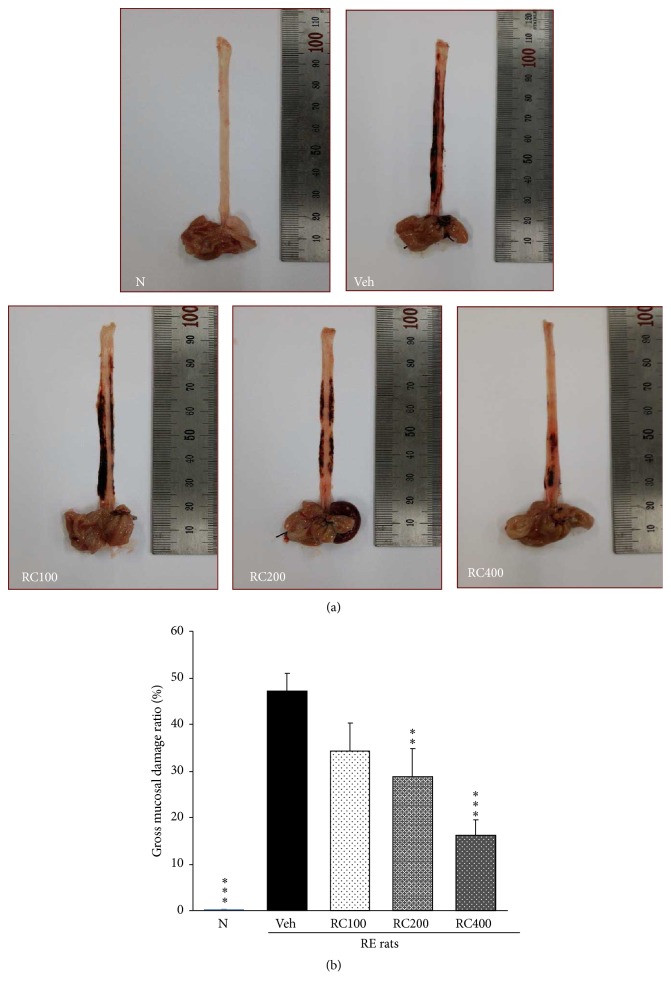
Gross evaluation of the esophageal mucosal damage. (a) Representative microphotographs of the esophagus. Esophageal lesion observed in rats with induced reflux esophagitis (RE) was ameliorated by RC-mix (100, 200, and 400 mg/kg body weight/day, p.o.) administration. (b) Gross mucosal injury ratio at the end of experiment. The gross mucosal injury was increased in RE rats compared with normal rats, but RC-mix administration led to a significant decrease (RC200, *p* < 0.01; RC400, *p* < 0.001). N, normal rats; Veh, positive control rats with reflux esophagitis (RE); RC100, RC200, and RC400 RE rats, animals treated with RC-mix 100 mg/kg, RC-mix 200 mg/kg, and RC-mix 400 mg/kg body weight, respectively. Data are the means ± SEM. Significance: ^*∗*^ *p* < 0.05, ^*∗∗*^ *p* < 0.01, and ^*∗∗∗*^ *p* < 0.001 versus RE control rat values. *n* = 6 in each group.

Figure 3(a) shows the results of the morphological examination of the esophagus. Morphological changes such as hyperemia and multiple erosions were observed in the rats with reflux esophagitis and damage to the normal rats was not apparent. The oral administration of RC-mix led to a marked decrease of gross mucosal damage in a dose-dependent manner. Accordingly, gross mucosal injury ratio in RE rats significantly increased compared with normal rats, but RC200 and RC400 pretreatment led to a significant decrease (*p* < 0.01, *p* < 0.001, resp.) (Figure 3(b)).

### 3.4. The ROS and ONOO^−^ Levels* in the Serum*

**Figure 4:**
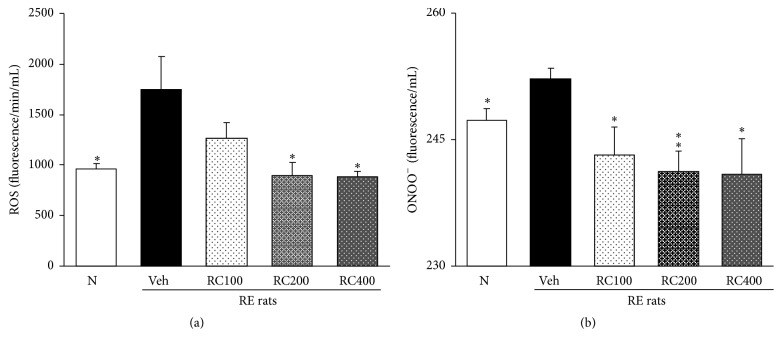
Effect of RC-mix on serum ROS and ONOO^−^ production in rats with induced reflux esophagitis (RE). N, normal rats; Veh, positive control rats with reflux esophagitis (RE); RC100, RC200, and RC400 RE rats, animals treated with RC-mix 100 mg/kg, RC-mix 200 mg/kg, and RC-mix 400 mg/kg body weight, respectively. Data are the means ± SEM. Significance: ^*∗*^ *p* < 0.05, ^*∗∗*^ *p* < 0.01 versus RE control rat values. *n* = 6 in each group.

As shown in Figure 4, the levels of ROS and ONOO^−^ in the serum in RE control rats were markedly higher than those of normal rats, whereas these enhanced levels were significantly inhibited by the administration of RC-mix. The reduced ROS level both RC200 and RC400 recovered nearly to those of normal (Figure 4(a)). The ONOO^−^ level in RC-mix treated experimental rats in comparison with RE control rats significantly decreased (Figure 4(b)). RC200 and RC400 treatment were superior to that of RC100. However, RC200 and RC400 showed a similar inhibitory effect.

### 3.5. Esophageal Nrf-2, HO-1, SOD, and Catalase Protein Expressions

**Figure 5:**
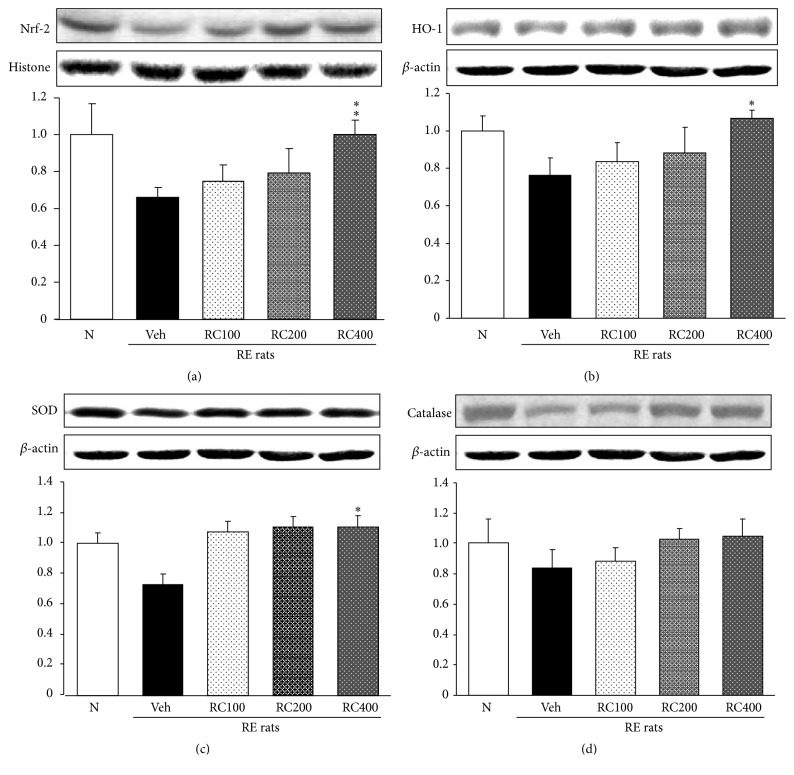
Esophageal Nrf-2 (a), HO-1 (b), SOD (c), and catalase (d) protein expressions. N, normal rats; Veh, positive control rats with reflux esophagitis (RE); RC100, RC200, and RC400 RE rats, animals treated with RC-mix 100 mg/kg, RC-mix 200 mg/kg, and RC-mix 400 mg/kg body weight, respectively. Data are the means ± SEM. Significance: ^*∗*^ *p* < 0.05, ^*∗∗*^ *p* < 0.01 versus RE control rat values. *n* = 6 in each group.

Figures 5(a) and 5(b) showed that esophageal expressions of Nrf-2 and HO-1 in RE control rats had tendency to decrease compared with those of normal rats. However, RC400 administration significantly regulated the nuclear Nrf-2 and cytosolic HO-1 expressions in the esophagus of reflux-induced esophagitis rats (*p* < 0.01, *p* < 0.05, resp.). These results suggest that induction of HO-1 may be through Nrf-2 activation. The protein expressions of SOD-1 and catalase were decreased in RE control rats compared with normal rats; however, RC-mix treatment led to upregulations of SOD and catalase (Figures 5(c) and 5(d)). Herein, SOD and HO-1 protein expressions in RC400-treated rats were increased significantly, whereas catalase showed a tendency to increase (without significance) in the esophagus.

### 3.6. Esophageal p-I*κ*B*α* and NF-*κ*Bp65 Protein Expressions

**Figure 6:**
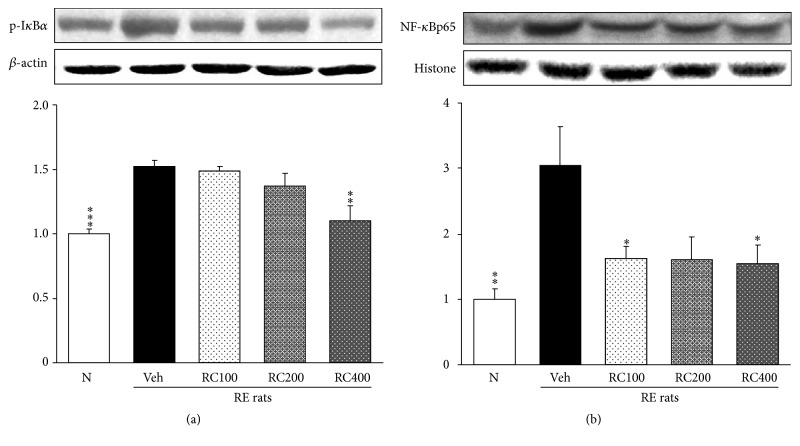
Esophageal p-I*κ*B*α* (a) and NF-*κ*Bp65 (b) protein expressions. N, normal rats; Veh, positive control rats with reflux esophagitis (RE); RC100, RC200, and RC400 RE rats, animals treated with RC-mix 100 mg/kg, RC-mix 200 mg/kg, and RC-mix 400 mg/kg body weight, respectively. Data are the means ± SEM. Significance: ^*∗*^ *p* < 0.05, ^*∗∗*^ *p* < 0.01, and ^*∗∗∗*^ *p* < 0.001 versus RE control rat values. *n* = 6 in each group.

As shown in Figure 6, the expression of p-I*κ*B*α* and NF-*κ*Bp65 proteins was analyzed by Western blot. The protein levels of p-I*κ*B*α*(Figure 6(a)) and NF-*κ*Bp65 (Figure 6(b)) increased in the esophagus of RE control rats, whereas these elevated levels significantly reduced in RC-mix treated rats with reflux-induced esophagitis. Particularly, p-I*κ*B*α* level was lowered nearly to that of normal rats by RC-mix 400 mg/kg treatment.

### 3.7. Esophageal COX-2, iNOS, TNF-*α*, and IL-6 Protein Expressions

**Figure 7:**
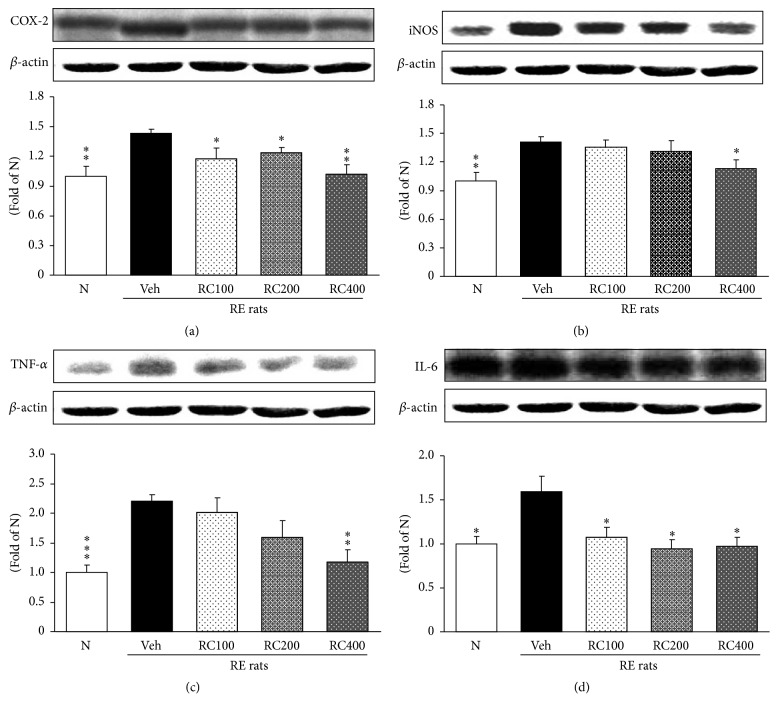
Esophageal COX-2 (a), iNOS (b), TNF-*α* (c), and IL-6 (d) protein expressions. N, normal rats; Veh, positive control rats with reflux esophagitis (RE); RC100, RC200, and RC400 RE rats, animals treated with RC-mix 100 mg/kg, RC-mix 200 mg/kg, and RC-mix 400 mg/kg body weight, respectively. Data are the means ± SEM. Significance: ^*∗*^ *p* < 0.05, ^*∗∗*^ *p* < 0.01, and ^*∗∗∗*^ *p* < 0.001 versus RE control rat values. *n* = 6 in each group.

The quantified COX-2, iNOS, TNF-*α*, and IL-6 protein expressions are shown in Figures 7(a), 7(b), 7(c), and 7(d), respectively. The inflammation-related protein expressions in the RE control rats were significantly augmented in the esophagus compared with normal rats. However, treatment with RC-mix suppressed these proteins in the esophagus. Moreover, COX-2 and IL-6 decreased significantly in all RC experimental rats and, above all, the administration of RC400 reduced nearly to normal levels or below in the esophagus. iNOS and TNF-*α* protein expression exhibited no significant changes in RC100 and RC200 but RC400 reduced significantly.

## 4. Discussion

Despite noticeable advances in modern medicine, reflux esophagitis (RE) remains one of worldwide problems with a considerable impact on quality of life and healthcare costs [27]. The etiology of RE is complex and various rather than a single cause, including hypersensitivity of the esophageal mucosa to physiological reflux, reduced mucosal defense mechanisms, and gastric motility disturbances [28]. It is generally shown that refluxate containing acid, pepsin, and bile causes inflammation, ulceration, and destruction of the normal squamous epithelium of the esophagus [29]. Synthetic drugs used in the treatment of RE are inadequate and sometimes can have serious side effects. In view of the undesirable side effects of synthetic agents, the treatment of RE is focused on traditional herbal medicines. In the present report, we explored the potential of Rhei rhizoma and* Coptis chinensis* mixture on experimentally induced reflux esophagitis in rats.

Rhei rhizoma is a famous traditional Korean medicine and used for treating many diseases, such as liver injury, gastrointestinal disease, constipation, and ulcers. The major antigastritis and antipeptic ulcer active constituents of Rhei rhizoma were sennoside A, emodin, aloe-emodin, chrysophanol, and rhein [30, 31]. Coptidis rhizoma has various pharmacological properties, such as gastroprotective, antidiabetic, hypolipidemic, analgesic, and neuroprotective effect [32, 33]. The major active compounds are alkaloids, including berberine, coptisine, palmatine, jatrorrhizine, and their chemical structures [34]. Particularly, berberine has been widely reported to improve reflux esophagitis, gastroenteritis, diarrhea, and colitis with few side effects [35, 36]. However, the mechanisms underlying the effects of Rhei rhizoma and Coptidis rhizoma mixture have yet to be investigated in an experimental model of reflux esophagitis. Therefore, the present study was conducted using an experimental reflux esophagitis model. The analyses of RC-mix have shown that berberine was the highest alkaloid present. Our previous study has reported that berberine could protect the esophageal mucosal damage in reflux-induced esophagitis by suppressing proinflammatory cytokines [37]. DPPH and ABTS radical scavenging assays, based on hydrogen atom transfer and electron transfer reaction together, have demonstrated that* in vitro* analytical methods are reliable determination of antioxidant activity of biological sample [38]. The results of the* in vitro* antioxidant assays (DPPH and ABTS, Figure 2) showed that the administration of RC-mix could improve RE-induced oxidative stress in the esophagus of rats.

The general pathophysiology of gastric disorders including RE has focused on the imbalance between offensive and defensive factors. The esophageal mucosa, through its preepithelial (mucus and bicarbonate ion), epithelial (epithelial cells), and deep postepithelial (blood vessels) mechanisms, represents one of the important defense mechanisms [39]. The preepithelial superficial defense mechanism is weak, so esophageal epithelial cells are easily exposed to refluxed contents and the prolonged contact with acid and pepsin can lead to morphological changes of esophageal tissues, including dilation of intracellular spaces, extensive erosion of esophageal mucosa, detachment of epithelial layer, and mucosal degeneration [40]. In the present study, the esophageal lesion score in RE control rats was remarkably increased compared with that of normal rats as already demonstrated by other authors [7]. However, the lesion score was significantly attenuated in both RC200 and RC400 animal groups, suggesting the potential therapeutic effect of RC-mix against RE.

The action of oxidative stress in inflammation-based GI tract diseases was widely known and, in the development of RE, it also results to be more important than the injuries caused by gastric acids [8, 41]. An imbalance in the generation of free radicals like ROS and reactive nitrogen species such as ONOO^−^ (which is highly toxic) was found to be responsible for the esophageal tissue damage; this finding was supported by the studies showing that tissue damage could be prevented with the use of antioxidant agent [42]. ROS causes oxidative damage in cellular components such as DNA, proteins, and membrane lipids. Overproduction of ROS results in oxidative stress, which leads to oxidative damage in cells by altering the structure of biomacromolecules, and this process has been implicated in a number of diseases. Therefore, the reduction of intracellular ROS may help prevent the onset and progression of diseases* via* protection of vital molecules [43]. Inducible nitric oxide synthase (iNOS) is a Ca^2+^-dependent cytosolic enzyme that forms nitric oxide (NO) from l-arginine, and NO reacts with the free radical superoxide (O^2−^) to form the toxic free radical peroxynitrite (ONOO^−^). Free radicals such as ONOO^−^ and O^2−^ damage cellular membranes and intracellular proteins, enzymes, and DNA. Elevated ONOO^−^, a powerful oxidant, produces oxidative stress, an imbalance between oxidants and antioxidants [44]. In the present study, the induced esophageal reflux determined the esophagus tissue damage, which was combined to an increase of ROS production. However, the RC-mix treatment was able to reduce the oxidative stress by significantly reducing the ROS production.

Nrf-2 is usually present within the cytosol; however, oxidative stress induces the translocation of Nrf-2 into nucleus. Nrf-2 leads to the transcription of antioxidant enzyme including SOD, catalase, and HO-1 by binding to antioxidant response element (ARE) [45]. Nrf-2-dependent genes, including SOD, catalase, and HO-1, are very important for cellular defense against oxidative stress and proper management of free iron is increased [46]. SOD interacts with O_2_^−^ to form H_2_O_2_, which is subsequently catabolised by catalase to H_2_O. HO-1 degrades heme to carbon monoxide (CO), biliverdin, and iron. The cytoprotection by HO-1 is mediated by various different mechanisms, including the catabolism of prooxidant heme to the antioxidant bile pigments biliverdin and bilirubin; the coordinate induction of ferritin, which chelates free iron; and the liberation of CO, which exerts meaningful anti-inflammatory and antiapoptotic effects [47]. Moreover, recent study suggests that HO-1 plays a critical role including cytoprotective and antioxidant and antiapoptotic activities in inflammatory diseases of the upper (esophagus and stomach) and lower (intestine) gastrointestinal tract [48]. In this study, the oral administration of RC-mix tended to increase SOD and catalase activities in a dose-dependent manner and the level of SOD became significantly higher in RC400 treated animals. In addition, the induced reflux esophagitis rats showed a decreased expression of HO-1 in esophageal tissues compared with normal rats, whereas the RC-mix administration resulted in a significant upregulation of HO-1 in RC400 rats. This suggests that the RC-mix treatment may effectively scavenge oxyradicals during the oxidative stress induced by the induced reflux esophagitis.

Our previous study [20] showed that NF-*κ*B pathway plays a key role in impairment of esophageal barrier function due to exposure to the gastroesophageal refluxate and regulates the transcription of a wide variety of genes involved in the inflammatory and immune response [49]. The NF-*κ*B dimer is sequestered in the cytoplasm of most cells through interaction with the inhibitory I*κ*B*α* protein and the inhibition of I*κ*B*α* phosphorylation leads to stabilization of NF-*κ*B/I*κ*B*α* interaction thus preventing nuclear translocation of NF-*κ*B [50]. The phosphorylation of I*κ*B*α* results in the translocation of NF-*κ*B and it means NF-*κ*B activation. A variety of stimuli can trigger NF-*κ*B activation such as infections, inflammatory cytokines, ultraviolet irradiation, and oxidative stress. The results of the present study show that RC-mix at a dose of 400 mg/kg body weight seems to reduce the phosphorylation of I*κ*B*α* and therefore could prevent the translocation of NF-*κ*B in esophageal tissue. Namely, the administration of RC400 significantly suppressed NF-*κ*B activation through the marked inhibition of I*κ*B*α* phosphorylation.

NF-*κ*B regulates the expression of inducible enzymes including COX-2 and iNOS and promotes the transcription of target genes such as TNF-*α* and IL-6. The role of COX-2 was augmented under inflammatory conditions, such as reflux esophagitis and Barrett's esophagus [51], and iNOS, which generates nitric oxide (NO), is an important mediator of reflux-induced cell signaling in esophageal cells. The upregulation of iNOS expression results in the overproduction of NO. NO reacts with O_2_^−^ and forms ONOO^−^. ONOO^−^ can directly cause DNA damage and participate in inflammation-related carcinogenesis [52]. When monocytes and macrophages are exposed to inflammatory stimuli, they secrete pleiotropic cytokines such as TNF-*α* and IL-6. TNF-*α* exerts multiple and potent proinflammatory activity, and its blockade results in profound downregulatory effects [53]. In addition, IL-6, which is a broad-spectrum cytokine with characteristics of an acute-phase reactant, has both proinflammatory and anti-inflammatory functions that affect processes ranging from immunity to tissue repair and metabolism [54]. In the present study, treatment with RC-mix in the reflux esophagitis model significantly decreased esophageal protein upregulation of NF-*κ*B related inflammatory mediators (COX-2 and iNOS). In addition, the protein expression of IL-6 was significantly downregulated following the administration of RC-mix. Whereas RC100 and RC200 did not significantly affect the expression of TNF-*α* the latter was significantly reduced by RC400 treatment.

**Figure 8:**
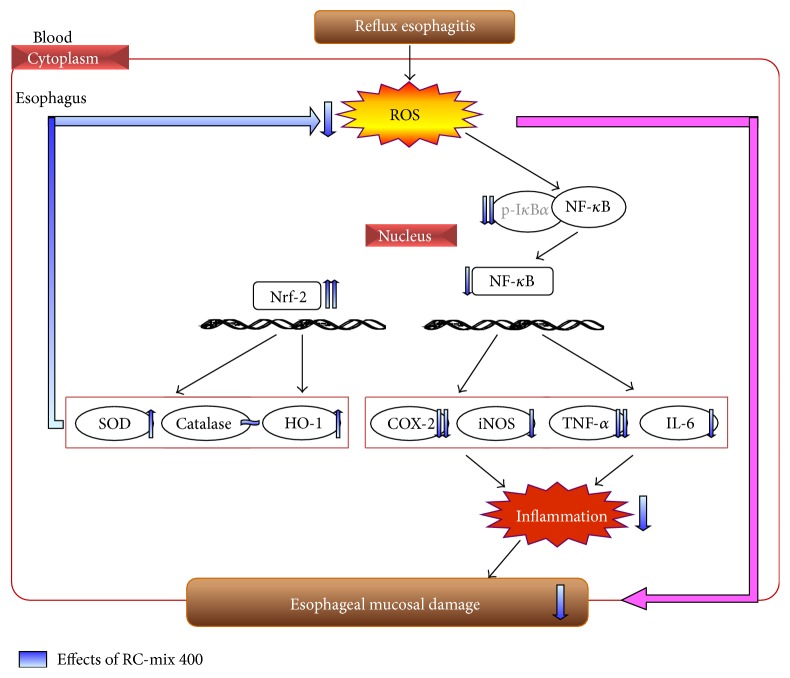
Predicted mechanism in esophageal tissue on administering RC400. RC400 decreased serum ROS and ONOO^−^ production. Further, RC400 ameliorated the values of proinflammatory mediators (COX-2 and iNOS) and cytokines (TNF-*α* and IL-6) regulated by NF-*κ*B and increased oxidative defense factor SOD and HO-1 proteins.

A recent study has shown that ROS are one of the potent factors in the pathogenesis of esophageal mucosal damage mediated by oxidative stress in an experimental model of reflux esophagitis [55]. In the present study, the administration of RC-mix reduced the oxidative stress biomarker and increased the activities of SOD and HO-1. Furthermore, the anti-inflammatory effects of RC-mix suggested that the inactivation of NF-*κ*B by blocking the phosphorylation of I*κ*B*α* led to the inhibition of the release of proinflammatory cytokines and mediators, reducing the inflammatory damage that is typical in the esophageal mucosa of rats with reflux esophagitis (Figure 8).

## 5. Conclusions

Rhei rhizoma and Coptidis rhizoma mixture includes the major flavonoids such as sennoside A, epiberberine, coptisine, palmatine, and berberine. RC-mix has a protective effect against esophageal mucosal damage in a dose-dependent manner. The RC-mix treated rats exhibited stronger anti-inflammatory activity through the elevation of antioxidant enzymes and the suppression of NF-*κ*B activation. Thus, the antioxidative abilities of RC-mix may potentially be used for the prevention of esophageal mucosal damage.
